# Effectiveness and safety of bifidobacteria and berberine in people with hyperglycemia: study protocol for a randomized controlled trial

**DOI:** 10.1186/s13063-018-2438-5

**Published:** 2018-01-26

**Authors:** Jie Ming, Shaoyong Xu, Chun Liu, Xiangyang Liu, Aihua Jia, Qiuhe Ji

**Affiliations:** Department of Endocrinology, Xijing Hospital, Fourth Military Medical University, 169 Changle Road West, Xi’an, 710032 China

**Keywords:** Bifidobacteria, Berberine, Hyperglycemia, Diabetes, Pre-diabetes, Chinese population, Randomized trials

## Abstract

**Background:**

Berberine is one of the most important examples of a Chinese traditional medicine that has hypoglycemic effects but there have been no randomized controlled trials of the drug in a larger sample. In addition, the use of probiotic biotherapy to maintain an appropriate intestinal flora may represent an effective early intervention for hyperglycemia. Unfortunately, there has been a shortage of relevant research on this possibility at the population level. This study was designed to determine the hypoglycemic effect and safety of both bifidobacteria and berberine administration to newly diagnosed patients with pre-diabetes or diabetes mellitus.

**Methods/design:**

This is a multicenter, double-blind, randomized, and parallel-controlled study that includes a run-in period of 2 weeks and a treatment period of 16 weeks, which will be conducted between June 2015 and October 2018. The 300 randomized patients will be assigned to the following four groups for 16 weeks’ treatment: *Bifidobacterium*, berberine, *Bifidobacterium* combined berberine, and placebo control groups. The primary outcome is the absolute value of fasting plasma glucose compared with baseline after 16 weeks of treatment.

**Discussion:**

This is the first randomized controlled trial to determine the hypoglycemic effect and safety of both bifidobacteria and berberine administration to newly diagnosed patients with pre-diabetes or diabetes mellitus. It may provide support for the use of berberine and bifidobacteria in the treatment of diabetes.

**Trial registration:**

ClinicalTrials.gov, ID: NCT03330184. Retrospectively registered on 18 October 2017.

**Electronic supplementary material:**

The online version of this article (doi:10.1186/s13063-018-2438-5) contains supplementary material, which is available to authorized users.

## Background

According to the latest national epidemiological survey of diabetes, China has prevalence rates of 9.7% and 15.5% for diabetes and pre-diabetes, respectively, among people of over 20 years of age [1], and it has become one of the countries with the largest number of diabetic patients. Oral hypoglycemic drugs have been widely used in China as the recommended first-line treatment, and these can effectively reduce blood glucose [2]. However, adverse reactions or side effects can be associated with their use, albeit benefits that outweigh the risks. For example, metformin can have gastrointestinal effects, sulfonylureas can cause hypoglycemia, and glucagon-like peptide-1 analogs can induce gastrointestinal reactions and antibody formation [3, 4]. Although insulin is relative effective and safe, it may also have risks of hypoglycemia, must be administered by injection and, therefore, its use is less straightforward. Therefore, it is necessary to develop novel effective oral hypoglycemic agents.

Chinese traditional medicine has been used for thousands of years. Diabetes and its treatment were described as early as the Wei and Jin periods (A.D. 220–420), and “Huanglian treats diabetes” was published in the *Renown Physicians’ Extra Records* [5]. With the modernization of traditional Chinese medicine, its use for the treatment of diabetes has attracted greater attention from researchers in recent years. Berberine is one of the most important examples of a traditional Chinese medicine that has hypoglycemic effects [6]. It has been used for the treatment of the complications of diabetes in China for 1500 years [7, 8]. Some studies reported that berberine can improve blood glucose and lipid levels in patients with type 2 diabetes mellitus and dyslipidemia [9–11]. A possible mechanism for its beneficial effects is an increase in the expression and activity of glucose transporter 1, thus increasing the glucose consumption of cells in a non-insulin dependent manner. In addition, it may reversibly inhibit insulin expression, as well as increasing insulin sensitivity at the level of the insulin receptor and activating the insulin signal transduction pathway [12–16].

In addition, because the intestinal tract represents the first organ to be exposed to glucose, and, therefore, has a key role in glucose metabolism, many researchers now believe that the composition of the intestinal flora is important in metabolic diseases such as type 2 diabetes [17–19]. An imbalance in the intestinal flora can result in the presence of deleterious metabolites in the body and cause metabolic endotoxemia, leading to an inflammatory response, the development of insulin resistance, and eventually type 2 diabetes [20]. However, probiotics, such as bifidobacteria, are protective against diabetes because they maintain balance in the intestinal flora [21, 22]. In addition, they may indirectly impact glucose metabolism through effects on intestinal hormones and/or the exocrine pancreas. Previous studies have shown that supplementation with bifidobacteria can improve glucose tolerance and glucose-induced insulin secretion by reducing endotoxemia and proinflammatory cytokine secretion by adipose tissue in high-fat-diet-induced diabetic mice, thus reducing inflammation and ameliorating the metabolic disorders associated with diabetes [22]. Therefore, the use of probiotic biotherapy to maintain an appropriate intestinal flora may represent an effective early intervention for diabetes. Unfortunately, there has been a shortage of relevant research on this possibility at the population level.

Thus, this study was designed to determine the hypoglycemic effect and safety of both bifidobacteria and berberine administration to newly diagnosed patients with pre-diabetes or diabetes mellitus, and their impact on the cardiovascular system, blood lipid metabolism, the intestinal flora, intestinal hormones, body weight, and quality of life.

## Methods/design

This is a multicenter, double-blind, randomized, and parallel-controlled study that includes a run-in period of 2 weeks and a treatment period of 16 weeks, which will be conducted between June 2015 and October 2018.

### Institutional setting

This multicenter study will be conducted in eight research centers in Xi’an, Shaanxi Province, China: the First Affiliated Hospital of the Fourth Military Medical University, the First Affiliated Hospital of Xi’an Jiaotong University, the Second Affiliated Hospital of Xi’an Jiaotong University, Shaanxi People’s Hospital, Xi’an Chang’an Hospital, Xi’an High-tech Hospital, Xi’an Central Hospital, and Shaanxi Aerospace Hospital.

### Research population and inclusion criteria

The inclusion and exclusion criteria are shown in the Appendix.

### Pharmacologic treatments

**Fig. 1:**
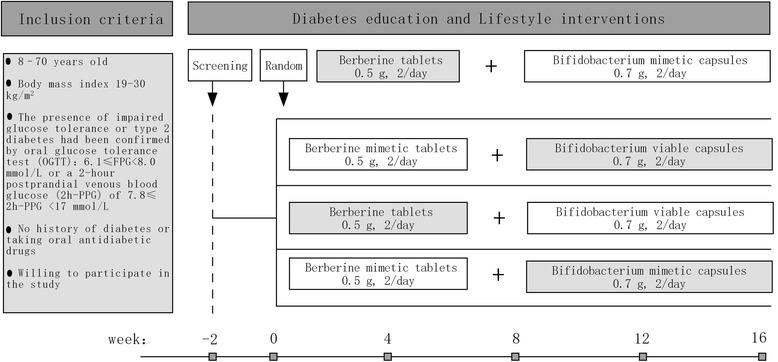
Inclusion criteria and interventions

The study will include an introductory period of 2 weeks and treatment for 16 weeks (Fig. 1). After the introductory period, the 300 successfully screened subjects will undergo baseline data acquisition and lifestyle guidance and be randomly assigned to the following four groups for 16 weeks’ treatment: *Bifidobacterium*, berberine, combination, and control groups.

The berberine group will receive berberine tablets, 0.5 g, two a day, and *Bifidobacterium* placebo capsules, 0.70 g, two a day, orally. The *Bifidobacterium* group will receive *Bifidobacterium* viable capsules, 0.70 g, two a day, and berberine placebo tablets, 0.5 g, two a day, orally. The combination group will receive *Bifidobacterium* viable capsules, 0.70 g, two a day, and berberine tablets, 0.5 g, two a day, orally. The control group will receive *Bifidobacterium* placebo capsules, 0.70 g, two a day, and berberine placebo tablets, 0.5 g, two a day, orally.

The preparations used will be berberine tablets (Northeast Pharmaceutical Group Shenyang First Pharmaceutical Co., Ltd., Lot No.: 130917), berberine tablet simulant (Northeast Pharmaceutical Group Shenyang First Pharmaceutical Co., Ltd., Lot No.: 20150101), *Bifidobacterium* viable capsules (Livzon Pharmaceutical Group, Lot No.: 20141013), and *Bifidobacterium* viable capsules simulant (Livzon Pharmaceutical Group, Lot No.: 141001).

During the study, researchers will administer other drugs that are not defined within the exclusion criteria and are necessary for the safety and health of the subjects to treat disease or discomfort. All of the drugs administered during the study will be recorded in the corresponding parts of the appropriate Case Report Form (CRF). Here, the specific type of drug (trade name or common name), indications for use, the date, and the dose used will be recorded. In addition, the following drugs will be banned or restricted during the study: any hypoglycemic therapy except for the study drugs, and systemic glucocorticoid (orally or intravenously administered), if used for more than seven consecutive days.

### Lifestyle interventions

Regulation of the diet and exercise of the subjects will be regarded as very important in this study, and discussion and implementation of a diet and exercise plan will be commenced in accordance with researchers’ suggestions from the beginning of the screening period to the end of the trial. A standardized meal suggestion (30 kcal/kg ideal weight/day, based on dietitian recommendations), will be provided, which includes 55% of calories from carbohydrate, 25% from fat, and 20% from protein, with these macronutrients being present in the respective proportions: 1/5, 2/5, and 2/5, and participants will be asked to be strict about their diet and to avoid short-term or high-intensity exercise. Researchers will emphasize the importance of diet and exercise to subjects at each treatment visit.

### Outcomes

The main and secondary outcomes are shown in Table 1.

**Table 1:** Primary and secondary outcomes of the trial

Purpose		Measurements
Primary outcome		The absolute value of fasting plasma glucose (FPG) compared with baseline after 16 weeks of treatment
Secondary outcomes	1	The proportion of patients who meet following blood glucose response: (1) HbA1c < 7.0%; (2) HbA1c < 7.0%, without severe hypoglycemia; (3) HbA1c < 7.0%, without diagnosed hypoglycemia; (4) HbA1c < 6.0%; (5) HbA1c < 6.0%, without severe hypoglycemia; (6) HbA1c < 6.0%, without diagnosed hypoglycemia; (7) FPG < 110 mg/dL (6.1 mmol/L); (8) FPG < 130 mg/dL (7.2 mmol/L)
	2	Changes compared with baseline: (1) 2-h postprandial glucose (2-h PPG): (2) glycosylated hemoglobin (HbA1c); (3) 2 h PPG < 140 mg/dL (7.8 mmol/L); (4) 2-h PPG <180 mg/dL (10.0 mmol/L); (5) HbA1c reduced by 0.5%; (6) blood pressure (BP); (7) lipid changes: total cholesterol (Total-C), low-density lipoprotein cholesterol (LDL-C), high-density lipoprotein cholesterol (HDL-C), and triglyceride (TG); (8) Body weight and Body Mass Index (BMI); (9) HOMA Index, insulin early phase and late-phase secretion index; (10) intestinal hormones (GLP-1, glucagon); (11) intestinal flora
Safety purpose		Laboratory data: electrocardiograph (ECG); pulse and blood pressure (BP); weight and physical examination.

*HbA1c* glycosylated hemoglobin

### Participant timeline

**Fig. 2:**
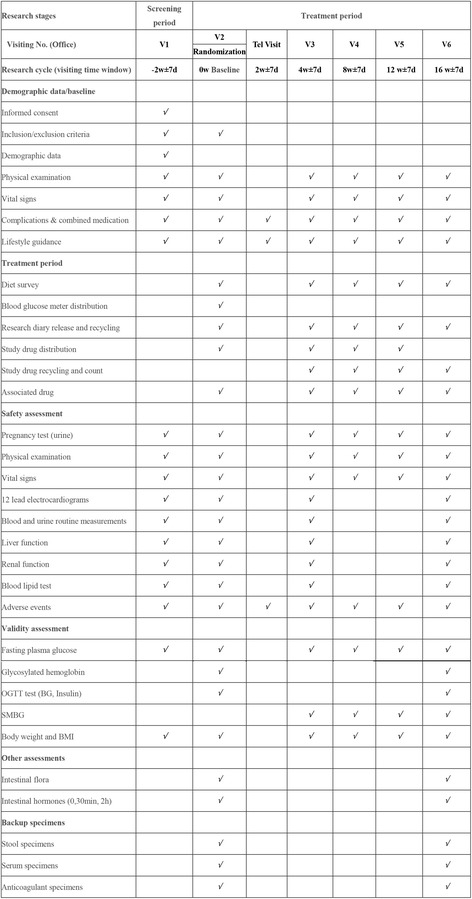
Standard Protocol Items: Recommendations for Interventional Trials (SPIRIT) Figure. Specific research plan and implementation steps. Note: Laboratory safety assessment assays were completed by sub-center laboratories, while the validity assessment laboratory assays and the intestinal indices were completed by the central laboratory. *OGTT* oral glucose tolerance test, *BG* blood glucose, *SMBG* self-monitoring of blood glucose, *BMI* Body Mass Index

The time course for participant registration, intervention, assessment, and follow-up is shown in the Standard Protocol Items: Recommendations for Interventional Trials (SPIRIT) Figure (Fig. 2).

### Randomization

The stratified blocked randomization method will be used. Stratification will be conducted by each research center to select the appropriate segment length. The statistical software SAS 8.2 PROC PLAN will be used to generate a random code list for 300 subjects to receive specific treatments. All randomized grouping number segments will be sent to the research centers with the corresponding treatment kits. Researchers will then select kits with the same series number for the study according to the visit sequence.

### Blinding and unblinding procedures

Because the two research drugs have different specifications and appearance, double-blind dual simulation will be selected as the method of blinding. The test and placebo drugs will be provided by the coordinating center in identical internal and external packaging.

A two-level blinding design will be used, with the first level being by group (groups A, B, C, and D) according to case number, and the second level by treatment (berberine, *Bifidobacterium*, combination and control). The random code list will be created by a statistical agency, the two-level treatment codes will be sealed separately in duplicate, with a copy being kept by each of the statistical agency and the central coordinator, and the treatment code will be not opened during the study. The study would have been considered invalid if any treatment code disclosure arose from any non-prescribed circumstances and if it affected the objectivity of the data obtained from the study.

A two-phase unblinding method will be adopted. When all of the CRFs have been entered into the database, the first unblinding (identifying groups A, B, C, and D) will be carried out after the blind review, data locking, and statistical analysis plans will be confirmed. The second unblinding will be conducted when the statistical analysis report is completed, to identify subjects in the berberine, *Bifidobacterium*, combination and control groups.

Each coded test drug will have a corresponding emergency letter, describing the subject’s medication, treatment methods, and the organization to which requests for emergency unblinding should be immediately reported. Emergency letters will be sealed and issued to the research center with the corresponding numbers, where they are preserved and not opened unless necessary. In the case of an emergency (for example, a severe adverse reaction), when the type of medication being administered must be known for the effective treatment of the subject, the researcher could access the appropriate letter. In this instance the identity of the opener, the opening date, and the reasons for doing so will be recorded on the CRF. Once unblinded, the subjects will withdraw from the trial, and be treated as expulsion cases. All emergency letters and CRFs will be collected at end of the trial for blind review.

### Data collection

Laboratory parameters will be used for validity and safety assessments (see Fig. 2 for details). The central laboratory will provide all the appropriate materials needed by the research centers for sample collection, processing, packaging, and transportation. Before starting the clinical study, a laboratory manual will be provided to center researchers, containing a detailed description. Insulin, glucagon, and GLP-1 will be measured by the central laboratory, while other assays are carried out by the sub-centers, according to the unified standard provided by the coordinator.

Before blood collection, subjects will sit for 5 min. A tourniquet will be used for up to 2 min. For serum insulin assay, 5 ml whole blood will be taken while fasting, and 30 min and 2 h after glucose intake. This will be centrifuged for 2 h, and the separated serum will be collected into cryopreservation tubes. For glycosylated hemoglobin (HbA1c) measurement 2 ml whole blood will be obtained and placed into blood collection tubes containing EDTA. For GLP-1 and glucagon assay, 5 ml blood will be obtained using vacuum blood collection tubes containing EDTA and aprotinin, after fasting, and 30 min and 2 h after a meal, and the centrifuged serum will be collected into cryopreserved tubes for storage at − 20 °C. Stool specimens the size of soybeans will be placed into dry, sterile collection tubes, and sent to the analysis center within 24 h for cryopreservation.

Prior to undergoing an oral glucose tolerance test (OGTT) subjects will consume a normal diet (with carbohydrate content no less than 150 g) for at least 3 days. After 10–12 h of fasting, they will be required to empty their bladder and an initial blood sample will be obtained. Then they will drink 75 g anhydrous glucose dissolved in 300 ml water over approximately 5 min, and the time of ingestion will be recorded. Further blood samples will be then obtained after 30 min and 2 h.

### Statistical methods

Because no previous studies of treatment with bifidobacteria have been conducted, this represents a pilot study. Sample size was estimated using NCSS PASS 11 (NCSS LLC, Kaysville, UT, USA) software, and it was planned that 300 subjects would be enrolled. The detailed statistical methods are described below.

Superiority hypothesis: in case of occurrence of superiority of either the berberine, *Bifidobacterium* or combination group, the test will be deemed successful, with the bilateral test level for α = 5% (no correction), and for β = 20% (power 80%).

The primary observation indicator in this study was the absolute value of fasting plasma glucose (FPG) compared with baseline after 16 weeks of treatment. The berberine sample size was estimated with reference to the STOP-NIDDM study [4] and Yifei Zhang’s study [11] on the use of berberine to treat type 2 diabetes with hyperlipidemia. In this study, berberine treatment decreased FPG by 1.45 ± 0.85 (standard deviation (SD)) mmol/L, while FPG in the placebo control group decreased by 0.6 ± 1.2 mmol/L. With a difference in FPG between the berberine and placebo groups of 0.85 mmol/L, the estimated pooled variance was 2, and the estimated sample size was 44.

The bifidobacteria sample size was estimated based on a decrease in FPG of 1.2 ± 1.1 mmol/L, and a decrease in FPG in the placebo control group of 0.6 ± 1.0 mmol/L. Assuming that the difference in FPG decrease between the berberine and placebo groups was 0.6 mmol/L, and the estimated pooled variance was 2, the estimated sample size was 88 persons in a single group. Allowing for some expulsions, the final sample size for each group was determined to be 50 for the berberine group, 100 for the *Bifidobacterium* group, 50 for the combination group, and 100 for the placebo control group, giving a total sample size of 300.

The primary analysis population will undergo at least one trial drug treatment and have at least one follow-up record comprising a full analysis set (FAS) and will be analyzed on an intention-to-treat (ITT) basis, including all randomized patients. All support analyses will be based on per-protocol sets (PPSs), and the security endpoint analysis will be based on a safety set (SS). The study will not incorporate a planned mid-point analysis.

All data will be analyzed using SAS 9.3 (SAS institute Inc, Cary, NC, USA) software using pre-programmed algorithms. Quantitative indices recorded will include the mean ± SD, median, maximum and minimum values; qualitative or grade indices will be recorded using a frequency distribution table. Two-sided tests will be used in all cases, and *P* ≤ 0.05 will be considered to be statistically significant. Fisher’s exact probability test will be used to compare the expulsion rates between the groups.

Validity analysis will be based on the FAS, using a covariance analysis model, with the main and secondary efficacy index baseline values being the covariates in the model, and the central effect serving as the fixed-effect term. The least squares estimates will be used to calculate the corrected mean and the 95% confidence intervals for changes in each group, and the 95% confidence interval of the Dunnett corrected mean difference between the test and placebo groups will be used to fit a covariance analysis model with central and group interactions to test the consistency among study centers. The applicability of this covariance analysis model will be investigated at the level of α = 0.10. Unless otherwise specified, the method of Carrying Forward (last observation carried forward, LOCF) will be used for sensitivity analysis of each validity analysis.

Security analysis will be based on a SS. Safety endpoints will be analyzed and adverse events aggregated. The continuous variables or frequency counts and percentage in safety and tolerability endpoints will be analyzed using descriptive statistics. The relative indices for hypoglycemic events and changes in weight will be compared between groups according to general principles.

## Discussion

In recent years, many researchers have continued to explore applications of traditional Chinese medicine and the regulation of the intestinal flora for the treatment of diabetes. Berberine and bifidobacteria are important examples of Chinese traditional medicine and probiotics, but a well-designed, randomized, double-blind clinical trial has not been conducted to date with sufficient sample size to validate these pre-clinical findings.

Many pre-clinical studies have shown that berberine acts at the cellular metabolic level, especially in hepatocytes, pancreatic beta cells, and adipocytes. Regulation of AMP-dependent protein kinase (AMPK) plays a central role in berberine’s effect on cellular pathways. A study found that berberine can increase insulin secretion and reduce antioxidant stress through this pathway [23], while another study showed that berberine can increase insulin receptor (InsR) expression, which it was shown to do in a time and dose-dependent manner in human hepatocyte cells. Berberine administration in the presence or absence of calcium phosphate protein C (a protein kinase C (PKC) inhibitor) or U0126 (an extracellular signal-regulated kinase inhibitor) and others demonstrated that berberine activates the *InsR* gene promoter mainly through activation of the PKC pathway and thus promotes expression of the *InsR* gene [24].

Clinical studies have also suggested that berberine is an effective therapy for diabetes and metabolic syndrome. The study of 31 cases by Yin et al. in 2008 found that berberine can reduce fasting and postprandial blood glucose by > 30% [25]. In the meta-analysis conducted by Dong et al. in 2012, 138 patients taking berberine (0.5–1.5 g/day, 8–12 weeks) and 133 taking placebo were included, and the results showed that berberine can reduce fasting plasma glucose by 12%, postprandial glucose by 16%, and glycosylated hemoglobin by 0.9% [26]. However, the sample sizes of the independent studies were small.

The intestinal flora has been proposed to be a human “microbial organ,” which has a number of important roles in the body. Bifidobacteria are symbionts that play an important role in the regulation of the intestinal flora. Cani et al. showed that bifidobacteria improve glucose tolerance and glucose-induced insulin secretion, and reduce high-fat-diet-induced endotoxemia and serum proinflammatory cytokine levels in diabetic mice, thereby ameliorating inflammation in the mice and improving their metabolic status [22]. However, the exact relationship between intestinal bifidobacteria and type 2 diabetes is not clear. There have been fewer studies on the differences in intestinal tract bifidobacteria between type 2 diabetes patients and healthy individuals, with no robust evidence to support a difference in the population.

This is the first large-scale, multicenter, double-blind, randomized, parallel-controlled study on berberine and bifidobacteria in China, with the objective of quantifying their hypoglycemic effects and safety in newly diagnosed cases of pre-diabetes or diabetes mellitus. It may provide support for the use of berberine and bifidobacteria in the treatment of diabetes.

### Trial status

The trial is currently recruiting participants. The recruitment began in June 2015 and is anticipated to end in October 2018. Trial registration number: NCT03330184. Registration date: 18 October 2017 – retrospectively registered. Registered at ClinicalTrials.gov; the URL of the trial registry record is https://register.clinicaltrials.gov/. The trials protocol was reported according to the SPIRIT statement (Additional file 1).

## Appendix

*Inclusion criteria*

1. Age between 17 and 70 years
2. Body Mass Index of 19–30 kg/m^2^
3. The presence of impaired glucose tolerance or type 2 diabetes had been confirmed by oral glucose tolerance test. (The V1 stage is defined by fasting plasma glucose (FPG): 5.6 ≤ FPG < 8.0 mmol/L, while the V2 stage is defined by 6.1 ≤ FPG < 8.0 mmol/L or a 2-h postprandial venous blood glucose (2-h PPG) of 7.8 ≤ 2-h PPG < 17 mmol/L (each sub-center laboratory))
4. Having not participated in any other clinical trial within the prior 3 months
5. Having voluntarily given their informed consent

*Exclusion criteria*

1. Type 1 diabetes mellitus
2. Diabetes patients with previously treated or untreated FBG ≥ 8 mmol/L or 2-h PPG ≥ 17 mmol/L
3. Women of childbearing potential who are pregnant, breastfeeding or intend to become pregnant or are not using adequate contraceptive methods
4. Impaired liver function, defined as aspartate aminotransferase (AST) or alanine transaminase (ALT) more than twice the upper limit of normal
5. Impaired renal function, defined as serum creatinine ≥ 133 μmol/L
6. Uncontrolled treated/untreated severe hypertension (systolic blood pressure ≥ 160 mmHg and/or diastolic blood pressure ≥ 95 mmHg)
7. Chronic gastrointestinal diseases
8. Cancer and medical history of cancer (except basal cell skin cancer or squamous cell skin cancer)
9. Any clinically significant disease or disorder which, in the investigator’ opinion could interfere with the results of the trial
10. Mental incapacity, psychiatric disorder, unwillingness or language barriers precluding adequate understanding or co-operation, including subjects being unable to read and write
11. Known or suspected hypersensitivity to trial products or related products
12. Known or suspected abuse of alcohol, narcotics or illicit drugs

## Additional file

Additional file 1: SPIRIT 2013 Checklist: recommended items to address in a clinical trial protocol and related documents*. (DOCX 591 kb)

## Abbreviations

AMPK: AMP-dependent protein kinase
ARF: Case Report Form
FAS: Full analysis set
FPG: Fasting plasma glucose
GLP-1: Glucagon-like peptide
InsR: Insulin receptor
ITT: Intention-to-treat
LOCF: Last observation carried forward
OGTT: Oral glucose tolerance test
PKC: Protein kinase C
PPS: Per-protocol set
SD: Standard deviation
SS: Safety analysis set
