# Supplementary figures and images for: Effectiveness and safety of bifidobacteria and berberine in people with hyperglycemia: study protocol for a randomized controlled trial
Source: Trials. 2018 Jan 26;19:72. doi: 10.1186/s13063-018-2438-5 (PMC5787258; doi:10.1186/s13063-018-2438-5)

| 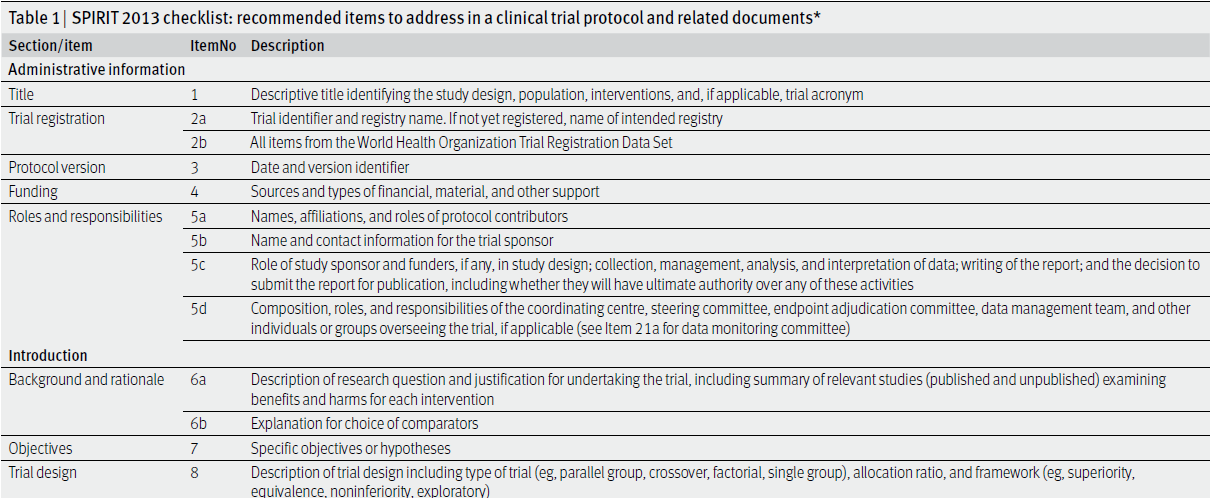 | Page |
| --- | --- |
|  |
| 1 |
| 2 |
| 2 |
| 13 |
| 12 |
| 12 |
| 12 |
| 12 |
|  |
|  |
| 3,4 |
| 3,4 |
| 4 |
| 4,5 |
|  |
| 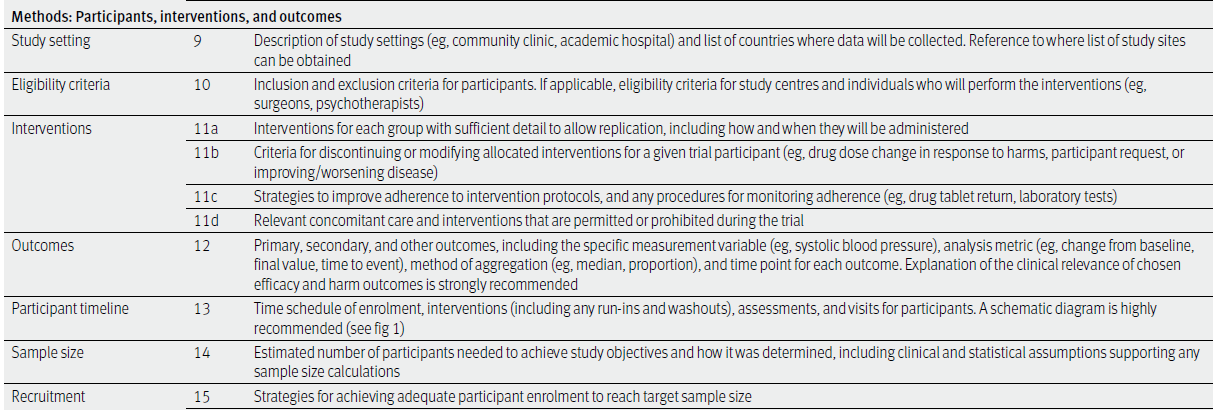 |  |
|  |
|  |
| 4,5 |
| 5, appendix 1 |
| 5 |
| Tab. 1 |
| SPIRITS fig. |
| 6 |
| 6 |
| 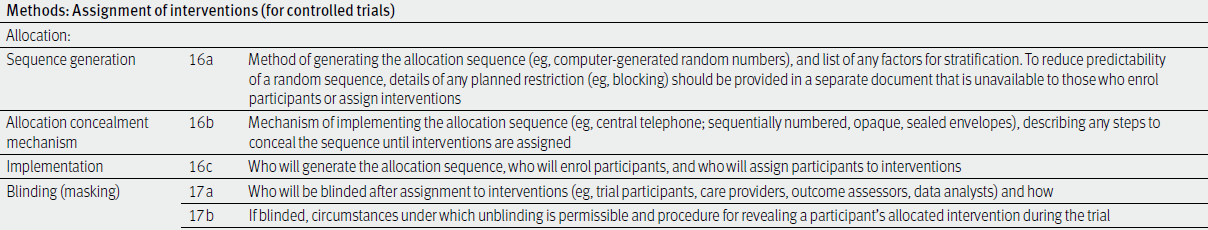 | 7 |
| 7 |
| 7 |
| 7 |
| 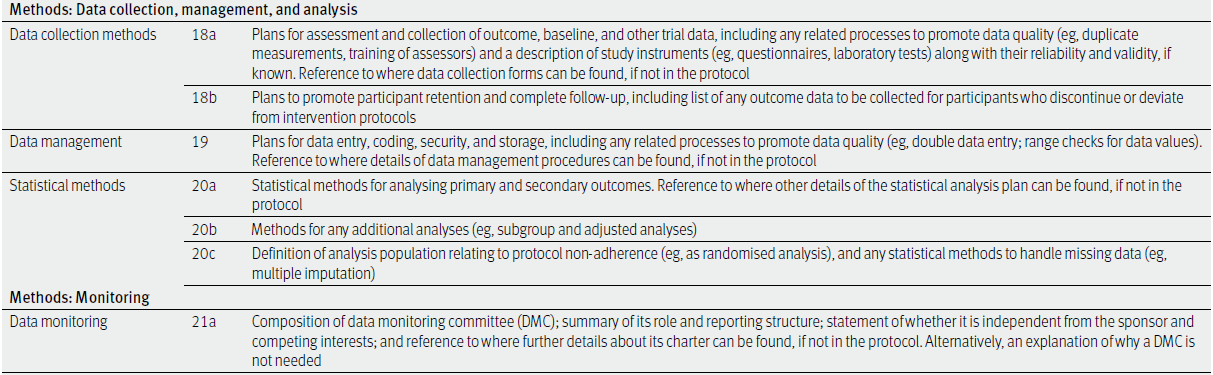 | 8 |
| 8 |
| 9,10 |
| 10 |
| 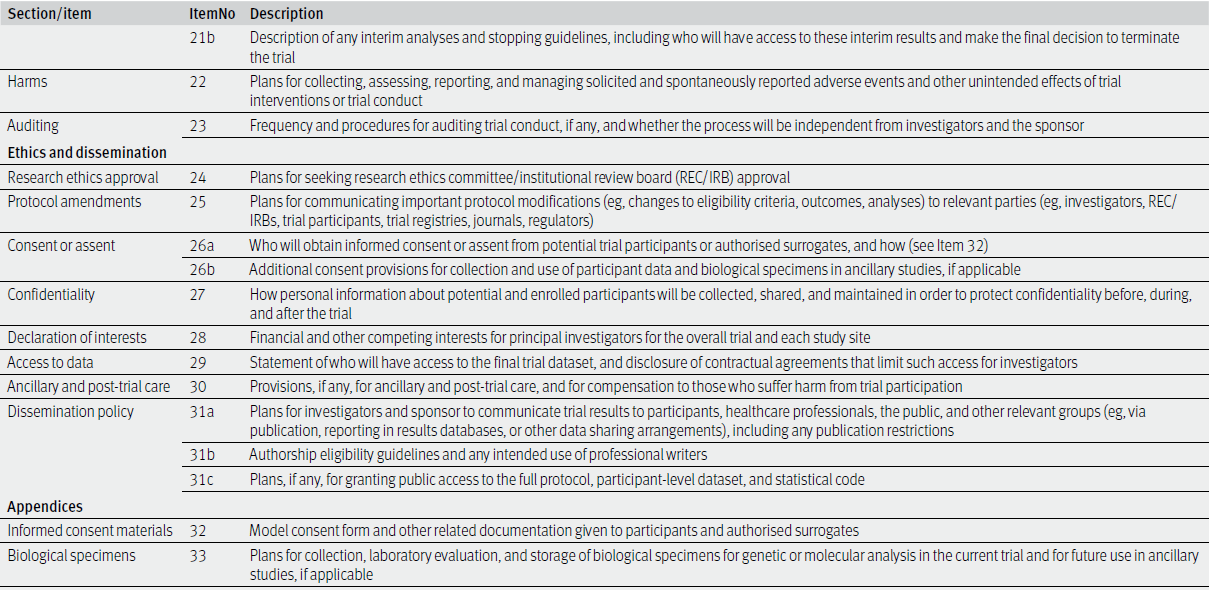 | 10 |
| 10 |
| 10 |
| 10 |
| 10 |
| 10 |
| 10 |
| 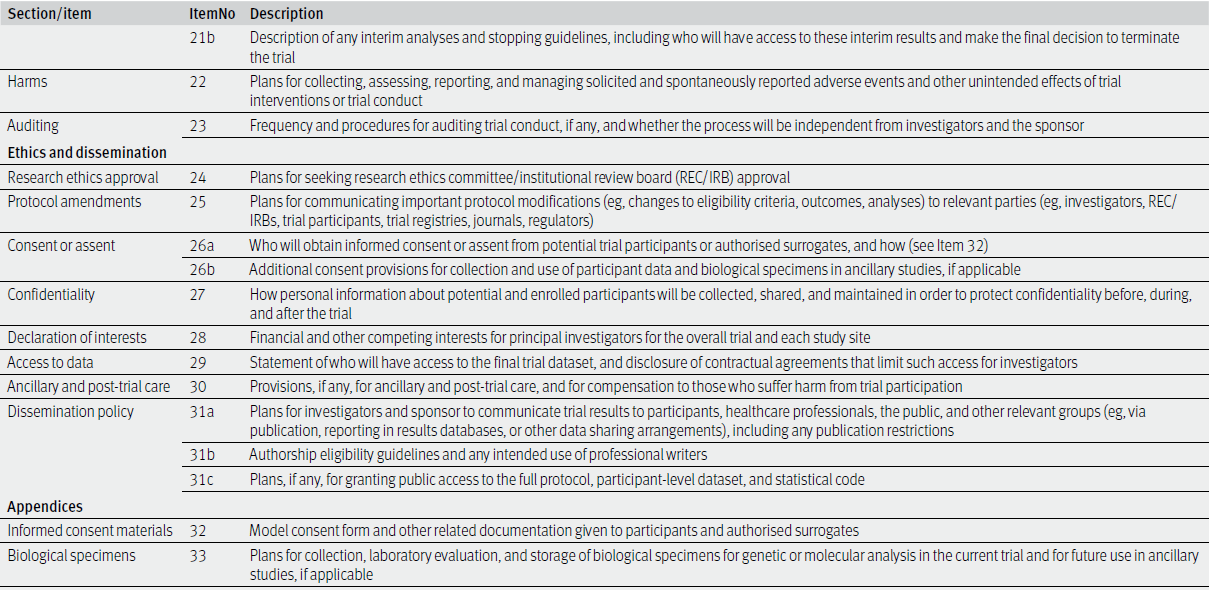 |  |
| 11 |
| 11 |
| 11 |
|  |
| none |
|  |  |

Supplement: Additional file 1: — SPIRIT 2013 Checklist: recommended items to address in a clinical trial protocol and related documents*. (DOCX 591 kb) [file 13063_2018_2438_MOESM1_ESM.docx]
